# Caracterisitiques des patients tuberculeux à l'ouest cameroun: 2000-2009

**DOI:** 10.11604/pamj.2013.16.39.2860

**Published:** 2013-10-05

**Authors:** Michel Noubom, Fabrice Djouma Nembot, Hubert Donfack, Patrick Stéphane Kouomboua Mfin, Floriane Tchasse

**Affiliations:** 1 Département des Sciences Biomédicales, Université de Dschang, Cameroun; 2 CDT de Baleng, Cameroun

**Keywords:** Tuberculose, Caractéristiques, Cameroun, Tuberculosis, Caracteristics, Cameroon

## Abstract

**Introduction:**

La tuberculose (TB) reste de nos jours un problème majeur de santé publique dans les pays en voie de développement. Elle devient de plus en plus importante à cause de l'infection au VIH. Cette étude avait pour but de caractériser les patients admis dans le plus grand Centre de Diagnostic et de Traitement de la Tuberculose (CDT) de l'Ouest Cameroun entre 2000 et 2009.

**Méthodes:**

Les patients de 15 ans et plus admis au CDT de Baleng durant la période allant du 1^er^ janvier 2000 au 31 décembre 2009 ont été inclus. Les données ont étés collectées grâce à une grille pré conçue. Le calcul des fréquences, moyennes et les comparaisons de groupes ont été faites pour ressortir les caractéristiques des participants.

**Résultats:**

2556 patients ont été inclus dans l’étude. 64,8% étaient de sexe masculin et l’âge médian étaient de 33ans. 2141 (83,7%) de patients présentaient une TPM+, 319 (12,5%) une TPM- et 96 (3,8%) une TEP. 64,7% des patients résidaient hors du district de santé d'implantation du CDT. 79,16% de patients tuberculeux ont fait le test de dépistage du VIH et la séroprévalence chez ceux testés était de 26,06%. Les différentes évolutions en fin de période de suivi de chaque patient ont été les suivantes: évolution favorable (guéri et traitement terminé) 1954(76,6%); perdus de vue 231(9,0%); décès 230(9,0%); transféré 92(3,6%); échec 49(1,9%).

**Conclusion:**

Une proportion considérable de patients résident loin du CDT ce qui augmenterait le perdus de vue et les transferts pendant le traitement. En plus vulgariser les autres CDT de la région, il est nécessaire de renforcer le système de transfert pour éviter les perdus de vue entre deux CDT.

## Introduction

La tuberculose reste un problème majeur de santé surtout en Afrique et plus particulièrement dans les pays sub-sahariens. En 2011, on estimait à plus de 8,7 million le nombre de nouveaux cas pour près de 1,5 million de décès dans le monde, ce qui fait de la tuberculose la deuxième cause de mortalité due aux maladies infectieuses dans le monde après le VIH/Sida [1]. Le fardeau causé par la tuberculose en Afrique est aggravé par l'infection au VIH. Tandis que l'infection au VIH fragilise le système immunitaire des patients et les rend susceptible à la tuberculose, ce dernier à son tour favorise la réplication [2] du virus ce qui aboutit à un cercle vicieux. Ainsi, le VIH alimente l’épidémie de la tuberculose dans les populations où ces deux infections coexistent. Au Cameroun, la prévalence du VIH chez les patients tuberculeux à bacilloscopie positive est passée de 16,6% en 1997 à 29,3% en 2007 [3, 4]. Vu le poids de ces deux infections et de la relation qui existent entre elles, l'OMS la préconise l'intégration de la lutte contre ces deux maladies [5]. Cette intégration s'est matérialisée au Cameroun en 2006 avec l'introduction dans le suivi des patients le test de dépistage du VIH. Plusieurs études sur la tuberculose ont étés faites au Cameroun mais la plus part s'est limité à l'Hôpital Jamot de Yaoundé et se sont déroulées pendant des périodes relativement courtes. Cette étude menée au CDT de Baleng et portant sur une période de dix ans a donc pour but de compléter les informations existantes sur la situation de la TB au Cameroun.

## Méthodes

### Site d’étude

Il s'agit d'une étude observationnelle rétrospective dans laquelle. Les données ont été collectées à partir des registres de prise en charge des patients admis au CDT de Lafé-Baleng du 1^er^ janvier 2000 au 31 décembre 2009. Les patients inclus dans l’étude sont ceux qui avaient au moins 15 ans au début de leur traitement.

L’étude s'est déroulé de mars à juin 2012 au CDT de Lafé-Baleng qui est l'un des plus anciens centres de traitement de la tuberculose au Cameroun. Il est situé dans la périphérie de la ville de Bafoussam, chef lieux de la Région de l'Ouest. Le CDT de Lafé-Baleng est rattaché au Centre Médical d'Arrondissement (CMA) de Baleng qui est l'une structure sanitaire publique du District de Santé de la Mifi. Cette structure hospitalière crée en 1935 reçois de nos jours en moyenne 550 patients par mois parmi lesquels un grand nombre de tuberculeux.

Au CDT de Lafé-Baleng comme dans tout le Cameroun, la prise en charge des patients tuberculeux se fait conformément aux directives de l'OMS [6] qui préconise la DirectlyObservedTreatment Short-Course strategy (DOTS), dont l'une des spécificités est le Traitement Observationnel Direct (DOT).

### Diagnostic et traitement de la tuberculose

Le patient suspecté de tuberculose pulmonaire produit trois échantillons d'expectorations qui seront analysés au microscope après coloration de Ziehl-Neelsen. Ceux suspectés de tuberculose extra pulmonaire seront diagnostiqués selon la localisation de la tuberculose. A la fin du diagnostic, les patients sont classés en trois groupes: conformément aux définitions de l'OMS: [4, 7]

- Tuberculose Pulmonaire à Microscopie positive (TPM+): présence d'au moins 1-9 BAAR (Bacille Acido-Alcoolo Résistant) dans au moins deux échantillons d'expectorations
- Tuberculose Pulmonaire à Microscopie négative (TPM&minus): si le patient présente des signes cliniques évocateurs de tuberculose pulmonaire malgré la négativité de trois examens de crachats et après la démarche suivante: dix jours de traitement par antibiotique non spécifique sans amélioration clinique suivi d'une persistance de la négativité de trois nouveaux examens de crachats sans cause évidente et une radiographie évocatrice d'une tuberculose pulmonaire.
- Tuberculose Extra Pulmonaire (TEP): si la tuberculose est localisé dans d'autres organes que les poumons.

Depuis 2006, il est systématiquement proposé aux patients tuberculeux le test du VIH. Ce test est effectué à l'aide des Test de Diagnostic Rapide Determine^®^ HIV½ (Abbotlaboratories, Tokyo, Japan) et Immunocomb^®^ II HIV1et 2 Bispot (Organics, Courbevoie, France) pour la confirmation.

Le traitement de la tuberculose est fonction du type de malade. Ainsi, pour les nouveaux cas, le traitement dur six mois soit deux mois de traitement intensif à la Rifampicine (R), l'Isoniazide (H), l'Ethambutol (E), et le Pyrazinamide (Z) suivie de quatre mois de continuation à la RH (2RHEZ/4RH). Pour les cas de retraitement, la Streptomycine (S) est introduite et le schéma thérapeutique se résume comme suit: deux mois de RHEZS, un mois de RHEZ et cinq mois de RHE (2RHEZS/1RHEZ/5RHE). Les patients co-infectés VIH tuberculose, reçoivent en plus le cotrimoxazole et les Anti Retro Viraux (ARV).

L’évolution des patients en fin de période de suivi est définie selon les normes de l'OMS [8] et adoptées par le Programme National de Lutte contre la Tuberculose. Ainsi, l’évolution favorable concerne les patients qui ont été guéri ou qui ont terminés leur traitement anti tuberculeux. Les cas de décès sont les patients qui sont décédés pendant le traitement indépendamment de la cause du décès. Les cas d’échec sont les patients TPM+ qui après la période de traitement produisent encore les expectorations positives à la coloration de Zeihl-Neelsen. Les cas d'abandon du traitement sont les patients qui ont interrompu leur traitement anti tuberculeux pendant deux mois et plus.

### Gestion et analyse de données

La collecte des données s'est déroulée du 05 avril au 19 août 2012. Les données collectées portaient sur le sexe, l’âge, le lieu de résidence, le type de malade (nouveaux cas, retraitement, transféré, rechute), le type de tuberculose, le type de traitement reçu, le statut sérologique VIH, la prise des ARV et du cotrimoxazole, les résultats d’ examens de crachat à 2/3, 5, et 6/8 mois, et l’évolution des patients. Les données ont été saisies à l'aide du logiciel Excel puis importées dans ledans le logiciel Epi Info version 3.5.3 (CDC) pour analyse. L'analyse a été faite par le calcul des fréquences et moyennes. Pour la comparaison des groupes, les tests de chi-2 (variables qualitatives) et de Student (variables quantitatives) ont été utilisés. La valeur de P utilisé dans l'analyse des données a été fixé à 0,05.

## Résultats

**Figure 1:**
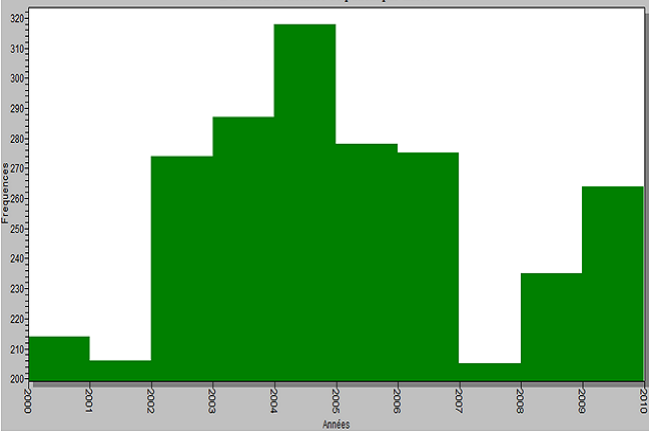
Evolution du nombre de patient admis au CDT de Lafé-Baleng pour tuberculose 2000-2009

Entre le 1^er^ janvier 2000 et le 31 décembre 2009, 2556 patients de 15 ans et plus ont été admis au CDT de Lafé-Baleng pour tuberculose (Figure 1). 64,8% étaient de sexe masculin et l’âge médian étaient de 33ans (intervalle interquartile 26-43 ans).

Les fréquences formes cliniques présentées étaient 2141 (83,7%) pour TPM+, 319 (12,5%) pour TPM- et 96 (3,8%) pour TEP. 1655 (64,7%) résidaient hors du District de Santé de la Mifi et parmi ceux-ci, 1014 (39,7%) venaient des Régions du Centre et du Littoral. Concernant le type de malades on avait 2264 (88,58%) de nouveaux, 97 (3,79%) de rechute, 92 (3,60%) de reprise traitement, 58 (2,27%) de transféré, 12 (0,47%) d’échec et 33 (1,29%) n’étaient pas notés dans le registre (Tableau 1).

**Tableau 1:** Caractéristiques générales des patients tuberculeux admis au CDT de Lafé-Baleng : 2000-2009

Caractéristiques	Homme(n,%)	Femme(n,%)	Total(n,%)
**Types de malades à l'entrée**			
Echec	6(0.4)	6(0.7)	12(0.5)
Nouveau	1457(87.9)	807(89.8)	2264(88.6)
Pas noté	22(1.3)	11(1.2)	33(1.3)
Rechute	66(4.0)	31(3.4)	97(3.8)
Reprise traitement	72(4.3)	20(2.2)	92(3.6)
Transféré	34(2.1)	24(2.7)	58(2.3)
**Diagnostic clinique**			
TPM-	207(12.5)	121(13.5)	328(12.8)
TEP	44(2.7)	40(4.4)	84(3.3)
TPM +	1406(84.9)	738(82.1)	2144(83.9)
**Lieu de résidence des patients**			
District de santé Mifi	555(33.5)	346(38.5)	901(35.3)
Région de l'Ouest (District de santé Mifi exclus)	345(20.8)	236(26.3)	581(22.7)
Région du Centre	157(9.5)	66(7.3)	223(8.7)
Région du Littoral	561(33.9)	230(25.6)	791(30.9)
Autre	39(2.4)	21(2.3)	60(2.3)
**Test du VIH**			
Fait	475(28.7)	300(33.4)	775(30.3)
Non fait	1182(71.3)	599(66.6)	1781(69.7)
**Statut sérologique VIH**			
Douteux	5(1.1)	0(0)	5(0.6)
Négatif	368(77.5)	200(66.7)	568(73.3)
Positif	102(21.5)	100(33.3)	202(26.1)

A partir de 2006, date à laquelle a été introduit dans le suivi des patients tuberculeux le test du VIH, 775 (79,16%) de patients ont fait le test et 204 (20,84%) ne l'ont pas fait. Parmi les tests faits, on avait 568 (73,29%) négatifs, 202 (26,06%) positifs et 5 (0,65%) douteux. La moitié des 202 patients dépistés positivement au VIH étaient de sexe féminin et 35 (17,33%) d'entre eux étaient sous traitement aux ARV, 115 (56,93%) ne recevait pas les ARV, et 52 (25,74%) cas n’étaient pas notés. Par ailleurs, tous les patients testés positifs au VIH recevaient un traitement préventif au cotrimoxazole. Le développement d'une TPM- a été positivement et significativement associé au statut VIH positif RR = 2,79 (IC = 1,87-4,17) et au fait de ne pas avoir fait le test du VIH RR= 2,11 (1,35-3,27). Entre ceux qui ont fait le test du VIH et ceux qui ne l'on pas fait, il n'existe pas une différent significativement d’âge (35 vs 34, P = 0,06), de sexe (p= 0,89) et des lieux de résidence (P= 0,37).

Les évolutions des patients inclus dans l’étude à la fin de leur traitement sont répartie de la manière suivante: évolution favorable (guéri et traitement terminé) 1954 (76,6%); abandon/perdus de vue 231 (9,0%); décès 230 (9,0%); transféré: 92 (3,6%); échec: 49(1,9%) (Tableau 2).

**Tableau 2:** Evolutions des patients par sexe en fin de période de suivie

	Homme (n,%)	Femmes (n,%)	Total (n,%)
**Evolution favorable**	1224(73.9)	730(81.2)	1954(76.4)
**Décès**	146(8.8)	84(9.3)	230(9.0)
**Défaillant**	185(11.2)	46(5.1)	231(9.0)
**Echec**	29(1.8)	20(2.2)	49(1.9)
**Transfert**	73(4.4)	19(2.1)	92(3.6)
**Total**	1657(100.0)	899(100.0)	2556(100.0)

## Discussion

Sur les 2556 patients inclus dans l’étude, 64,8% étaient de sexe masculin et l’âge médian étaient de 33ans. Ce résultat montre apriori que les hommes sont plus touchés par la tuberculose que les femmes. Ce résultat suit la tendance observé à l'Hôpital Jamot de Yaoundé [8], l'un des Centres national de référence pour la prise en charge des patients tuberculeux au Cameroun [9]. Cette information se justifierait par une forte prévalence de fumeurs dans la population des hommes au Cameroun (12,7% contre 2,0% chez les femmes) [10], ceci en considérant qu'il est établi que le tabagisme est fortement associé au développement de la tuberculose [11, 12]. Toutes fois, cette information ne refléterait pas la réalité dans la population totale parce que selon l'OMS, deux fois plus de cas de tuberculoses sont notifiés chez les hommes que chez les femmes dans les pays en voie de développement ceci étant due en partie aux facteurs socioéconomiques et culturels qui limitent l'accessibilité des formations sanitaires aux femmes [13].

64,7% des patients résidaient hors du District de santé de la Mifi (où se trouve le CDT de Baleng). Ceci s'explique par le fait que le CDT de Baleng est un centre historique de traitement de la tuberculose. Ainsi, plusieurs personnes pensent que c'est le seul centre traitement de la tuberculose et certains se disent que la prise en charge y est mieux comparée à d'autres CDT. Cette attraction du CDT de Baleng est aussi due à sa capacité d'accueil. En effet, le CDT possède 20 lits consacrés uniquement à la prise en charge des patients tuberculeux. Ainsi, plusieurs autres CDT dont la capacité d'accueil est faible transfèrent plusieurs de leurs patients au CDT de Baleng. Par ailleurs, 39,7% des patients inclus dans l’étude résidaient dans les villes de Douala et de Yaoundé. Ceci s'explique par le fait que les patients tuberculeux se rapprochent de leurs localités d'origine pour associer traitement traditionnel et traitement moderne. En outre, les patients en phase avancées de la maladie préfèrent se rapprocher de leur village dans l'optique de réduire les dépenses funéraires en cas d’éventuel décès.

Entre 2006 et 2009, 204 soit 20,84% de patients n'ont pas fait le test de dépistage du VIH. Toutes fois, cette proportion régresse en fonction des années allant de 36% en 2006 à 15,53% en 2009. Ceci peut être mis au compte des différentes actions de sensibilisation de la population et de formation du personnel médical sur la prise en charge de la co morbidité tuberculose/VIH. Cette proportion reste néanmoins supérieure à celle observée à l'Hôpital Jamot de Yaoundé où elle était en 2009 de 13,8% [4].

26,06% des patients testés au VIH étaient positifs. Cette proportion est proche de 29,3%, valeur observé à Yaoundé en 2007 par les études de Kuabang et al. [3]. Cette valeur est très supérieure à la prévalence dans la population générale qui était en 2007 estimée à 5,1% [14]. La forte prévalence du VIH chez les patients tuberculeux est bien documentée [15–17]. En effet, l'infection au VIH favorise l'activation de la forme latente *Mycobacteriumtuberculosis* vers une infection tuberculeuse active tandis que *Mycobacteriumtuberculosisfavorise* la réplication du VIH précipitant ainsi la dépression immunitaire et le développement vers des formes sévères de la maladie [13, 18].

Le développement d'une TPM- était positivement et significativement associé au statut VIH positif OR = 2,79 (IC = 1,87-4,17). Cette observation est en conformité avec des études faites précédemment dans de nombreux pays [19, 20]. En Zambie par exemple, une étude faite sur 100 patients présentant une culture positive à la TB pulmonaire a montré que 24% chez les VIH négatifs présentaient des crachats négatifs contre 43% chez les patients VIH positifs [21].

Dans cette étude, le taux d’évolution favorable (guéri et traitement terminé) et le taux d'abandon en fin de période de suivi des patients sont respectivement de 76,6% et 9,0%. A l'Hôpital Jamot de Yaoundé, ces taux étaient respectivement de 68,1% et 20,1% en 2007 [4]. Selon El-C Julio Rakotonirina et al, l'indicateur spécifique du programme de lutte contre la tuberculose est le taux d'abandon ceci à cause de l'influence significative de l'infection à VIH sur les taux de décès et d’échec au traitement 22. En comparant le taux d'abandon du CDT de Lafé-Baleng et celui de l'hôpital Jamot de Yaoundé (centre national de référence dans la prise en charge de la tuberculose), on peut penser que le programme de lutte contre la tuberculose fonctionne mieux au CDT de Baleng qu’à l'Hôpital Jamot de Yaoundé. Ceci ne reste qu'une hypothèse car plusieurs facteurs de confusions peuvent exister et ceux-ci peuvent se recruter parmi les caractéristiques socio démographiques et économiques des patients.

Cette étude nous a permis ressortir les caractéristiques des patients tuberculeux admis au CDT de Baleng sur 10 ans (2000-2009). Comme toutes études, ce travail comporte des limites. Le fait qu'on a travaillé sur les données secondaires ne nous a pas permis de contrôler la qualité des données présents dans les registres de prise en charge des patients.

## Conclusion

Une proportion considérable de patients admis au CDT réside hors du District de Santé de la Mifi, ce qui augmenterait le taux de transfert durant le traitement. Il faut de ce fait non seulement améliorer le système de transfert des patients pour éviter les perdus de vue entre deux CDT mais aussi vulgariser les autres CDT de la région pour que les patients prennent leur traitement dans le CDT le plus proche de son lieu de résidence. La prévalence du VIH chez les patients tuberculeux reste très élevée. Il est donc nécessaire de renforcer l'intégration de la prise en charge de ces deux pathologies afin de réduire le taux de mortalité due à ces maladies.
